# The Use of Artificial Neural Networks to Forecast the Behavior of Agent-Based Models of Pathophysiology: An Example Utilizing an Agent-Based Model of Sepsis

**DOI:** 10.3389/fphys.2021.716434

**Published:** 2021-10-14

**Authors:** Dale Larie, Gary An, R. Chase Cockrell

**Affiliations:** Department of Surgery, Larner College of Medicine, University of Vermont, Burlington, VT, United States

**Keywords:** agent-based model (ABM), machine learning, sepsis, neural networks, time series

## Abstract

**Introduction:** Disease states are being characterized at finer and finer levels of resolution *via* biomarker or gene expression profiles, while at the same time. Machine learning (ML) is increasingly used to analyze and potentially classify or predict the behavior of biological systems based on such characterization. As ML applications are extremely data-intensive, given the relative sparsity of biomedical data sets ML training of artificial neural networks (ANNs) often require the use of synthetic training data. Agent-based models (ABMs) that incorporate known biological mechanisms and their associated stochastic properties are a potential means of generating synthetic data. Herein we present an example of ML used to train an artificial neural network (ANN) as a surrogate system used to predict the time evolution of an ABM focusing on the clinical condition of sepsis.

**Methods:** The disease trajectories for clinical sepsis, in terms of temporal cytokine and phenotypic dynamics, can be interpreted as a random dynamical system. The Innate Immune Response Agent-based Model (IIRABM) is a well-established model that utilizes known cellular and molecular rules to simulate disease trajectories corresponding to clinical sepsis. We have utilized two distinct neural network architectures, Long Short-Term Memory and Multi-Layer Perceptron, to take a time sequence of five measurements of eleven IIRABM simulated serum cytokine concentrations as input and to return both the future cytokine trajectories as well as an aggregate metric representing the patient’s state of health.

**Results:** The ANNs predicted model trajectories with the expected amount of error, due to stochasticity in the simulation, and recognizing that the mapping from a specific cytokine profile to a state-of-health is not unique. The Multi-Layer Perceptron neural network, generated predictions with a more accurate forecasted trajectory cone.

**Discussion:** This work serves as a proof-of-concept for the use of ANNs to predict disease progression in sepsis as represented by an ABM. The findings demonstrate that multicellular systems with intrinsic stochasticity can be approximated with an ANN, but that forecasting a specific trajectory of the system requires sequential updating of the system state to provide a rolling forecast horizon.

## Background

The characterization of the gene expression or protein level patterns associated with clinical disease, which generally manifest as physiological derangements, has led to attempts to use this type of fine-grained, detailed information to forecast clinical outcomes. This approach underlies the concepts of personalized and precision medicine, where disease characterization in terms of molecular-level features (microstates) are intended to more finely define and distinguish patients who might have otherwise similar physiology (macrostates). Increasingly, machine learning (ML) has been investigated as a means of aiding in the ability to predict and forecast the course of disease. Modern ML generally involves training an artificial neural network (ANN) on a given data set such that the ANN “learns” an underlying function that generates the data. While a powerful method, ML-trained ANNs can be brittle and prone to overfitting, which can lead to their failure when applied in real-world situations (Ross and Swetlitz, 2017; Strickland, 2019; D’Amour et al., 2020). As ML is extremely data-intensive, training is often augmented by the use of synthetic data; however, it is crucial that the generated surrogate/synthetic data effectively replicates the underlying generative process of the real-world system being learned. This issue is less important for such static tasks such as image recognition/classification but takes on considerable importance if time-series/dynamic processes (and therefore functions) are being analyzed. The need to effectively generate synthetic data is accentuated in biomedical applications, where, in general, biomedical data sets are relatively sparse, particularly in terms of time series data needed to predict or forecast the dynamic course of disease. This sparsity is further accentuated when molecular-level biomarker panels are proposed as the means of disease characterization, as currently this information can only be acquired through invasive blood sampling. Thus, there is an inherent tension between the desire for a finer-grained characterization of disease state and limitations in terms of both availability of such data and the ability to correlate these detailed representations to the physiological derangements present clinically.

Multi-scale simulation models that represent cellular and molecular mechanisms can reproduce the dynamics of tissue or system level physiology and pathophysiology have potential as a means of generating synthetic training data, but these models come with their own challenges and limitations. Specifically, dealing with the high-dimensional parameter spaces of such complex mechanism-based models presents computational challenges in terms of calibration and validation. Additional ML methods have been proposed as an adjunct to the exploration of these models’ high dimensional parameter spaces (Cockrell et al., 2019; Ozik et al., 2019; Wang et al., 2019), including the training of artificial neural networks (ANNs) as surrogates for the mechanism-based model (Wang et al., 2019). However, to our knowledge, the application of ML to train ANN surrogates for agent-based models (ABMs), a prevalent method for multi-scale computational modeling, has not been previously reported in the biomedical literature. This is a potentially significant capability, as ABMs structurally share many of the features of biological systems (Bonabeau, 2002; An et al., 2009; Metzcar et al., 2019) and exhibit behaviors not necessarily represented by other types of modeling methods, particularly in terms of their stochastic behavior and reflection of biological heterogeneity (Cockrell and An, 2017). The ability of ABMs to generate “emergent” phenomena (Bonabeau, 2002), i.e., where populations of components and their interactions lead to system-level phenomenon that cannot be directly inferred from the behavioral rules governing the components is particularly relevant to being able to translate cellular and molecular mechanisms and data into system-level behavior manifesting as physiology. Cell-based ABMs explicitly represent existing knowledge about cellular and molecular mechanisms, which is the level at which modern medicine strives to characterize patients in a precise and personalized fashion (e.g., biomarker or -omics panels), and through their simulation are able to generate aggregated, system-level output corresponding to the physiology at which disease primarily manifests. Given the prevailing interest in characterizing disease states through molecular-level profiling and the application of ML methods to forecasting the physiological trajectories of disease, we believe that it is important to examine the capabilities and limitations of applying ML to forecast trajectories that bridge microstate (mediator/molecular profiles) and macrostate (system-level/physiological output). Toward that end we present herein an investigation of the ability of trained ANNs to forecast the dynamic behavior of a complex biomedical ABM used to simulate acute systemic inflammation and the clinical condition of sepsis.

### The Multi-Scale Challenge of Sepsis

Sepsis is a complex physiological and clinically significant problem with approximately 1 million cases in the United States each year, with a mortality rate between 28–50% (Wood and Angus, 2004). Sepsis is a highly dynamic process with multi-scale features, ranging from clinical phenotypes characterized by features such as multi-system organ failure, down to the molecular level with dysregulation of the body’s internal cytokine signaling network (Cockrell and An, 2017, 2019, 2021). While care process improvements in the treatment of sepsis, such as the development of treatment bundles and practice guidelines, have improved clinical outcomes in the past few decades, the search for new drugs to treat the biological-basis of sepsis has been marked by complete failure: there is currently not a single drug approved by the U.S. Food and Drug Administration that targets the underlying pathophysiology of sepsis (Angus, 2011; Buchman et al., 2016). One of the major challenges in designing therapies for sepsis is an inability to effectively forecast the disease trajectories of individual patients, thereby limiting the effective sub-stratification of this heterogeneous population into those biologically similar enough to control. Existing means of classifying sepsis patients, such as with the Sequential Organ Failure Score (SOFA; Vincent et al., 1996) or various biomarker panels (Gibot et al., 2012; Riedel, 2012; Samraj et al., 2013), while potentially useful for coarse-grained outcome risk stratification, are only able to provide population-level projections that cannot effectively be updated to an individual patient’s disease course. Adding to the limitations of data-centric population-based scoring systems is the inherent stochasticity of the biological processes driving sepsis. The presence of stochasticity in the system governing inflammation makes accurately predicting the entire trajectory of the disease, or accurately predicting the patient state 30 days into the future, given one point of assessment, an impossibility (see description of Stochastic Trajectory Analysis regarding sepsis in (Cockrell and An, 2017)).

### Biological Heterogeneity, Stochasticity, and Forecasting

Ultimately, the biological heterogeneity seen clinically is generated from a combination of inter-patient (genetic variability) and intra-patient (stochastic processes) effects. The result is that it is not tractable to comprehensively enumerate all possible biomarker states and configurations (i.e., phenotypes) that can be generated from a specific systemic perturbation or injury. The challenge (and solution) is similar to that faced by Q-Learning (Watkins and Dayan, 1992) (now Deep Reinforcement Learning); Q-learning is a type of reinforcement learning in which agents determine what action to take (*a*) by looking up their current state (*s*) in the lookup table, *Q(s,a)* that lists the probability of a desirable outcome based on that decision. Because the lookup table needs to provide this probability to guide the decision process it requires a finite (and computationally tractable) state space. In order to work effectively in continuous (infinite states) search spaces Q-learning utilizes the Universal Approximation Theorem (Barron, 1993), which states that a feed-forward neural network can approximate, to arbitrary fidelity, a real and continuous function. In the case of Q-learning, it is the lookup table that is being approximated; we note that the lookup table does not necessarily meet the strict mathematical definition for continuity, however, the technique works in practice as long as the density/granularity of the lookup table is sufficiently fine. Acquiring time series data of this granularity is often not logistically feasible, therefore we pose that mechanism-based simulations can serve as means of generating such surrogate data. In particular, given their structural similarity to biological systems, ABMs are appealing candidates for this task.

In previous work, we have demonstrated that the cytokine signaling network which controls the inflammatory process can be modeled as a random dynamical system (Cockrell and An, 2017, 2018), which is a system that evolves in time according to fixed rules, but also incorporates stochasticity (Bhattacharya and Majumdar, 2003; Arnold, 2013). Knowledge of the underlying cellular and molecular processes of acute inflammation has been used to create a dynamic model, the Innate Immune Response Agent-based Model (IIRABM; An, 2004), that can serve as a proxy model for the development of more advanced prediction and forecasting methods. The IIRABM is an ABM of the innate immune response that represents the endothelial-blood interface (e.g., the inside of blood vessels in the tissue region of interest) and the response of that system to either injury or infection. The IIRABM simulation is initiated with the application of a simulated injury or infection to the endothelium. The injury is defined by five parameters: injury size, microbial virulence, microbial toxigenesis, environmental toxicity, and host resilience. The simulated inflammatory response is generated by the damaged endothelium, and recruits a variety of inflammatory cells, including neutrophils, macrophages, and a suite of T-lymphocytes, to respond to, contain, and heal the injury/infection. The simulation then proceeds until it reaches a terminal state – either complete healing or death, which is triggered when the aggregate system damage exceeds 80%. This threshold has been chosen to represent the ability of supportive medical technology (i.e., a ventilator) to keep people alive in situations in which they would otherwise die.

Despite its acknowledged abstraction the IIRABM has proven useful in examining the complexity of sepsis and the challenges associated with trying to treat the syndrome. The IIRABM has been used to demonstrate the use of *in silico* clinical trials as a means of evaluating the plausibility of planned potential interventions (An, 2004), provided fundamental insights into the mathematical and dynamic properties of sepsis that account for patient heterogeneity (Cockrell and An, 2017), demonstrating the futility of standard biomarker-based outcome prediction (Cockrell and An, 2017), and served as a proxy model (An et al., 2017) for control discovery for sepsis. This most recent control discovery work has employed advanced computational methods such as genetic algorithms/evolutionary computing (Cockrell and An, 2018) and deep reinforcement learning/artificial intelligence (Petersen et al., 2019) to describe what would be required for multi-modal treatment of sepsis. While the IIRABM is nearly 20 years old its central component structure remains valid and has predicted a series of behaviors associated with sepsis that have since been recognized in the subsequent years, specifically the temporal concurrence of pro- and anti-inflammatory cytokine responses (as opposed to sequential pro- and compensatory responses) (Osuchowski et al., 2006; Tamayo et al., 2011) and the importance of the immunoparalyzed recovery phase of sepsis, particularly with respect to its prolonged duration (Ferguson et al., 1999; Boomer et al., 2011; Hotchkiss et al., 2013a, b). Key to all these studies is the recognition that even though the IIRABM is an abstract representation far less complex than the “real” immune system, it has structural properties that mimic a multicellular biological system (i.e., composed of semiautonomous components that harbor the system’s stochastic potential), and generates the type of system dynamics that challenges traditional methods of biomedical analysis. As such we consider the IIRABM a useful surrogate for generating biology-like synthetic data that bridges mediator-level microstate and system-level microstate output that can be used to examine the ability of ML to capture its behavior. The current work aims to train an ANN on simulated data generated from the IIRABM and evaluate its sufficiency as a surrogate for the IIRABM by assessing the ability of the trained ANN for dynamic trajectory prediction of the IIRABM.

## Materials and Methods

The foresting procedure is divided into two principal tasks: (1) predict future cytokine trajectories in an 11-dimensional space (microstate characterization); and (2) regress the overall “health” of the simulation as a function of its current cytokine profile (predicting system macrostate). Training and validation data was generated using the IIRABM (Cockrell and An, 2017). The training/validation set was composed of cytokine measurements for 11 unique cytokines over 10,000 time-steps in 66,000 *in silico* patients. Networks were constructed Using Keras (Gulli and Pal, 2017), a TensorFlow based deep learning library for Python.

### Trajectory Forecasting

In order to forecast future values in the cytokine time series, we utilized long short-term memory (LSTM) recursive neural networks (RNN). RNNs are different from standard multi-layer-perceptron networks because they have a neural network contained within a cell which takes information from the current input to help determine the adjusted state of the cell based on its current cell state. This adjusted cell state becomes the new cell state, and an output is determined for the network.

Long short-term memory networks’ memory cells have a unique structure, characterized by an input gate, two update layers, and an output gate to determine the adjusted cell state (Hochreiter and Schmidhuber, 1997). The memory cells in LSTM networks allow for more long term memory than typical RNNs which make them well suited for time-series analysis and prediction (Nelson et al., 2017). Noting this, an LSTM network will likely be able to predict future cytokine levels, given that they are continuous and previous cytokine levels will likely have a large impact on near-future values.

We constructed a unique network for each cytokine that was to be predicted; each LSTM network takes five sequential 11-dimensional cytokine profiles as input and predicts the subsequent value(s). The first three layers of the network are 100-node LSTM layers; the output from these layers are fed into two fully connected layers of 300 and 200 nodes, respectively, then to a single output node, resulting in 296,301 trainable parameters. Training data was arranged into five sequential 11-dimensional points as training input features and the next 11-dimensional point as the training label. The data was then shuffled to avoid biasing the training. After data preprocessing, 8,576,100 data sequences and labels were used to train the network. The loss metric used to train this network is mean absolute error (MAE), and the Adam optimizer (Kingma and Ba, 2014). Each network was trained until loss converged to a minimum.

For the ultimate utilization of this network, 11 cytokine values are observed for five time steps, then a prediction for each of the next values is made using its own LSTM network. This set of 11 observations is combined into one 11-dimensional point, which is then added to the original five samples as the next sample. Predictions are made recursively in this manner for 100 time steps after the initial observation. Accuracy of this algorithm was measured using the average MSE across the 11 cytokine values at 1, 2, 3, 4, 5, 10, 25, 50, and 100 time steps after the initial observation. Prediction variance and error bars were calculated through stochastic variations to the dropout layer (Baldi and Sadowski, 2013), as demonstrated with regards to Active Learning for regression in (Tsymbalov et al., 2018).

As a comparison of the efficacy of LSTM neural networks, MLP prediction networks for each cytokine were also created. Each network functionally acts the same as the LSTM networks, accepting five sequential 11-dimensional points in cytokine space and predicting the future value for a single cytokine. Each network has a structure beginning with a fully connected layer of 1000 nodes, followed by a function to flatten the output shape from a 5 by 1000 array to a single vector of length 5000. Next is another fully connected layer of 1000 nodes, then a 1% permanent dropout layer, feeding into a fully connected layer of 500 nodes, then another fully connected layer of 500 nodes, then finally to a single output node. These networks were trained using a loss function to minimize MSE. Eleven-dimensional cytokine profiles, predicted either from the LSTM network the MLP network are then fed into an MLP-regressor (described below) in order to translate the cytokine profile into an aggregate measure of patient health or disease state.

### Health Metric Regression

The IIRABM uses the “Oxygen Deficit” metric as a measure of health, where a low oxygen deficit is good, and a high oxygen deficit is bad; “Oxygen Deficit” is therefore the system-level output that corresponds to the macrostate of the IIRABM. We note that, both *in silico* and *in vivo*, cytokine profiles provide a non-unique mapping to state-of-health (a concept which is more nebulously defined *in vivo* than in our *in silico* model). As such, error is expected when attempting to regress from an 11-dimensional cytokine profile to a single health metric.

This regression was performed by using a fully connected deep network that takes an 11-dimensional cytokine vector as input, feeding into two fully connected layers with 1,500 nodes each, then into a layer with 150 nodes, and finally to a single output node. The loss metric used to train this algorithm is MSE. Using the regression network, a prediction of oxygen deficit trajectory can be made from the 11-dimensional matrix created by the LSTM network. Overall accuracy was measured by comparing the oxygen deficit path to the predicted path and calculating the MSE.

The prediction of whether an *in silico* patient will live or die, or the decision on whether or not pharmacologic therapeutics are more likely to be beneficial than detrimental, is ultimately based on the temporal trajectory of the patient’s state of health (in this case, a measure of systemic oxygen deficit). The predicted trajectory in 11-dimensional cytokine space is then fed into the health-metric regression network to forecast the most-likely outcome, time-to-outcome, and time-horizon for potentially effective therapeutic interventions.

Additionally, we created a multi-layer perceptron (MLP) to predict the future oxygen deficit trajectory as a function of past values only, effectively treating the simulation output as a Markov chain. This network expects an input of five sequential oxygen deficit values and will return a single future oxygen deficit value predicted for the next time step. The structure of this MLP begins with a fully connected layer of 1000 nodes, followed by a 1% permanent dropout layer, then two fully connected layers of 150 nodes each, followed by a single output node. This network was trained using a loss function to minimize MSE. Trajectory prediction for this network is made in the same recursive manner as the cytokine prediction networks.

## Results

**FIGURE 1:**
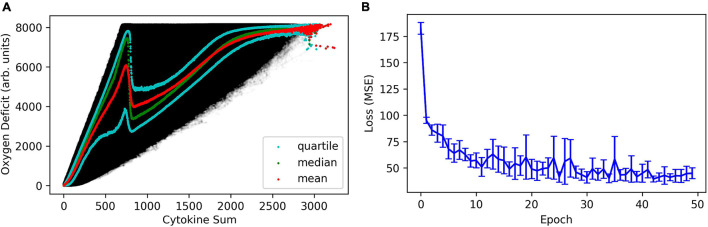
In panel **(A)**, we present the variance in oxygen deficit as a function of the sum of cytokine concentrations in the whole area of simulated tissue, effectively compressing an 11-dimensinal vector into a scalar quantity indicative of total biological activity (e.g., cells performing functions) in the simulated tissue. In panel **(B)**, we show the mean absolute error in the regression of the oxygen deficit as a function of cytokine profile, as a function of training epoch.

It is important to note that the map which translates a cytokine profile (microstate) into its associated oxygen deficit (macrostate; and vice-versa) is non-unique, and therefore some amount of error is expected and unavoidable. In Figure 1A, we present the variance in oxygen deficit as a function of the sum of cytokine concentrations in the whole area of simulated tissue. The sum of cytokine concentrations is a coarse metric that roughly represents the amount of inflammation (no distinction is made between pro- and anti-inflammatory signals) and inflammatory signaling present in the model. This is analogous to what is seen clinically – patients that see ostensibly identical insults/infections/injuries will invariably present a range of responses, in terms of temporal cytokine profiles or other clinical physiological observables (heart-rate, blood pressure, temperature, etc.).

This figure also illustrates a key difference between the structure of the noise in the IIRABM and the stochastic structure in a stochastic differential equation; the noise present in the IIRABM varies spatio-temporally and cannot be represented with a closed-form analytical expression. Very generally speaking, the reason for this is that when cell-signaling is high, there is lots of activity in the model, and therefore lots of opportunities for stochastic events. This can be illustrated with a simple thought experiment: consider two system states, one with a single infected cell and one with 10 infected cells, and each infected cell has a probability of infecting a single neighbor, and some probability of healing. If we evolve the simulation a single time step, system 1 can have 0, 1, or 2 infected cells, while the range of infected cells in system 2 can vary from 0 to 20 (depending on the spatial configuration of the infected cells). In Figure 1B, we show the mean absolute error as a function of training epoch when training the health regression neural net. The error quickly converges to a minimum with a relatively constant error of approximately 200 units (on a scale of 8160), with the caveat that the predicted error would be lower when the true oxygen deficit is lower, and higher when the true oxygen deficit is higher.

**FIGURE 2:**
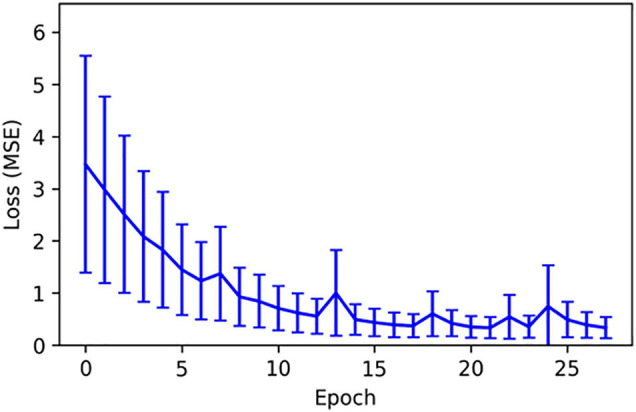
The mean squared error (in arbitrary units) as a function of training epoch for TNFα. The training of this cytokine prediction network was representative of all simulated cytokine prediction networks.

Cytokine trajectories present similar stochastic properties as the oxygen deficit: when levels are high, the plausible range of cytokine expression for the subsequent time step is larger than when levels are low. We present the mean squared error (in arbitrary units) as a function of training epoch for TNFα, which is representative of the full cytokine set in Figure 2. Once again, the network quickly converges to a low and constant level of error. We note that the total error quickly and significantly increases as we extend the time-prediction horizon past 100 time-steps. This distinguishes this methodology from that of ML-augmented surrogate modeling (Cicchese et al., 2017) because we do not claim the ability to accurately represent the entire course of a sepsis disease trajectory (up to 90 days in our computational model) using neural-network approximations.

**FIGURE 3:**
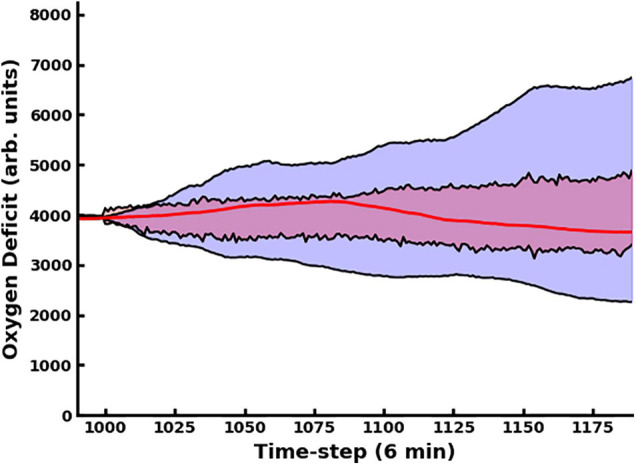
In this figure, we compare the dual-network predictor, which uses an MLP to predict the 11-dimensional cytokine profile trajectory and then another MLP to regress the oxygen deficit value (shaded in red), with an MLP which is informed solely by prior values for the oxygen deficit (shaded in blue).

**FIGURE 4:**
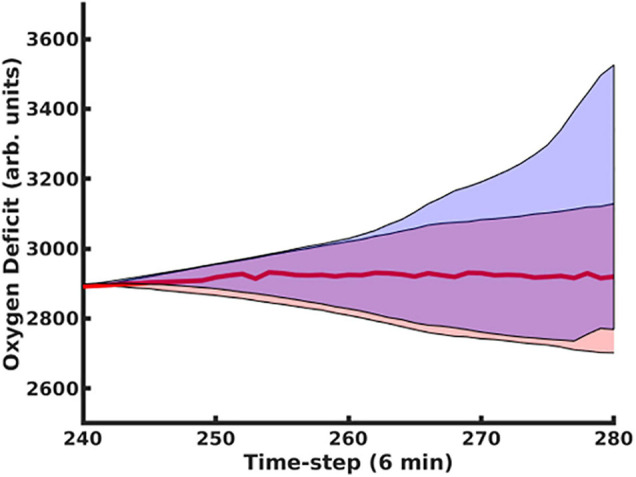
Here we present trajectory clouds for the MLP and LSTM-generated predictions. In both cases, 11-dimensional cytokine trajectories were predicted. The predicted values were then fed into the MLP regressor network, which regressed the future predicted oxygen deficit as a function of the predicted cytokine profile trajectories. The future health-trajectory probability cloud predicted using the MLP network is shaded in red; the future health-trajectory probability cloud predicted using the LSTM network is shaded in blue; the true trajectory is plotted in red.

**FIGURE 5:**
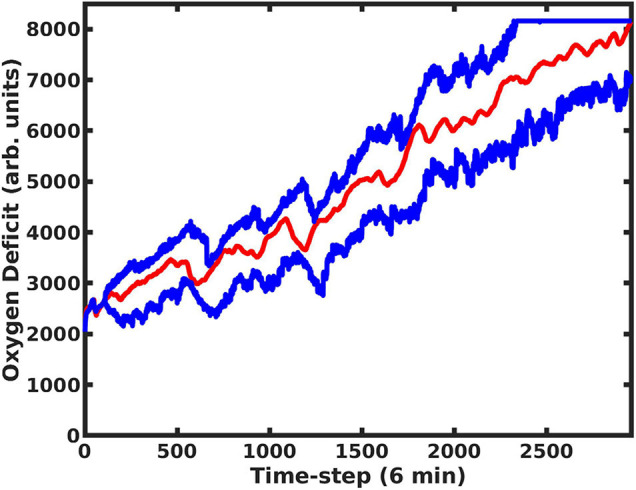
Using the MLP Trajectory Forecast Model combined with the MLP Oxygen-Deficit Regressor, we determined the upper and lower boundaries for the future health-trajectory probability cone, which are indicated by the blue lines; the actual trajectory is plotted with a red line. Predictions began at *t* = 200 and were update upon every time-step.

The use of the dropout layer allows for the simple creation of an ensemble of predictive networks by stochastically varying the specific node(s) in the layer that are dropped out, allowing us to visualize probability clouds for future trajectories. In Figure 3, we compare the dual-network predictor, which uses an MLP to predict the 11-dimensional cytokine profile trajectory and then another MLP to regress the oxygen deficit value (shaded in red), with an MLP which is informed solely by prior values for the oxygen deficit (shaded in blue); the performance of the dual-network model is significantly more stable and accurate when compared with the MLP using only the oxygen deficit for prediction. In Figure 4, we have visualized the probability cloud for future health trajectories generated using the MLP network (shaded in red), future health trajectories generated using the LSTM network (shaded in blue) and plotted the true trajectory (red line). This figure visualizes a single prediction iteration (predict future cytokine trajectory, regress state of health) for the above-described workflow. As new data is fed into the model about the true trajectory of the system, the forecast cloud is updated. The actual health trajectory typically lies in the center of the probability cloud, which is a clear benefit of the ensemble approach. In Figure 5, we display the probability cone for the entire simulation run, starting at the 240th time step, and then updating the trajectory cone on every subsequent time step.

**FIGURE 6:**
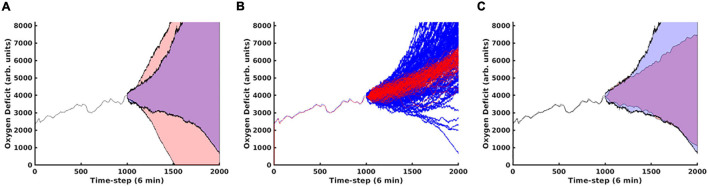
In this figure, we compare predictions generated using the MLP Cytokine Trajectory Prediction/MLP Oxygen-Deficit Regressor with a set of stochastic replicates generated by the simulation. In panel **(A)**, we contrast the future health-trajectory probability cones with networks that used data collected in the first 24 h (shaded in red) and networks that excluded the first-day data (shaded in blue); we note that the blue area appears purple as it is entirely contained within the red area. In panel **(B)**, we present 100 stochastic replicates of the actual simulation health-trajectory, reseeded at the time of the first prediction (red) compared with the predicted probability cone trajectories (blue). In panel **(C)**, we generated the simulation trajectory cone through reseeding the simulation’s random number generator at the upper and lower boundaries of the trajectory cone every 100 time steps from *t* = 1100 to *t* = 1800.

In Figure 6, Panel A, we contrast predictions that considered the full time evolution of the system when training the neural network model, shaded in red, with predictions that only used training data collected after the 240th time step, representing approximately 1 day. The network that only utilizes data collected more than 24 h post-injury performs substantially better. This is primarily due to the massive amount of stochasticity introduced at the time of injury; the degree of this stochasticity is significantly larger in magnitude than later in the simulation, as discussed below. In Panel B, we display the same oxygen-deficit probability cone as in Panel A, however, also reseed the simulation’s random number generator at this time step to generate 100 stochastic replicates of the time evolution of that specific instantiation of the IIRABM. We see that the predicted probability cone has a greater spread than the actual probability cone, however, we note that the MLP predictor is constantly updating its trajectory predictions: the set of observations $\left\{t_{-5}^{a},t_{-4}^{a},t_{-3}^{a},t_{-2}^{a},t_{-1}^{a}\right\}$, where $t_{-5}^{a}$ has the superscript, “a”, representing an actual observation, and the subscript, “−5” to denote that the time point is five points prior to the starting reference point, is used to predict $t_{0}^{p}$, where the superscript, “p”, indicates a predicted observation. Eventually, the set of points used to generate the prediction will consist entirely of previously predicted points, allowing for the compounding of any errors. In Panel C, we show the same probability cone as in Panel A, however, this time, we have re-seeded the simulation every 100 time steps at *t* = 1100 to *t* = 1800, for 100 stochastic replicates each. This is a more direct comparison since the MLP predictor effectively reseeds itself every time step. We observe that the actual probability cone is significantly wider than in Panel B, but still not as wide as the predicted cone. This is discussed in detail below.

## Discussion

The MLP predictor which predicts cytokine trajectories and uses those to regress the oxygen deficit performs better than using an LSTM to predict future state-of-health trajectories, however, this does *not* represent a failure of the LSTM method (or indicate superiority over the MLP). This is best illustrated in Figure 1, which illustrates the non-unique mapping between a specific cytokine profile and a physiological state of health, which is well-known clinically (Bergquist et al., 2019). The accuracy of the cytokine trajectory predictions, shown in Figure 2, is high, but even with an accurate prediction of the future cytokine profile, turning that profile into an informative state of health prediction is not possible. Additionally, this predictor also outperforms the NN model which used an MLP to predict future oxygen deficit from previous values of oxygen deficit, not incorporating any cytokine data (see Figure 3). This indicates that, while a static cytokine profile can be correlated with a wide range of health-state, the dynamics/trajectories of these mediators provide actional information regarding system state.

Additionally, we note that the predictor performs better when using training data starting 1 day after the simulated injury perturbs the system, and the reasoning for this is similar to that above, in that the cytokine and spatial dynamics are dominated by stochasticity. When the simulated injury occurs, a large, contiguous, area of tissue is injured with a homogenous injury, representing a significant perturbation to the system; thus, early simulation behaviors contain a significant amount of stochasticity, leading the training data to be less informative as to the true mechanisms which underlie the dynamics of the simulation.

Recognizing that there are configurations in which the system is more or less strongly influenced by randomness can also help to explain why treating the global simulation output as a Markov chain (or full cytokine trajectory output as a Markov random field, as we have described in (Cockrell and An, 2017)) is only an approximation. The full simulation, which takes place on a two-dimensional grid, is memoryless, and begins with a homogenous injury. However, as the injury evolves, the spatial distribution of damage or of various cytokine concentrations begins to vary, due to both stochastic and deterministic influences. All this information about spatial heterogeneity is lost when it is collapsed into a single trajectory. A Markov Transition Matrix (or kernel) could be constructed for the simulation output that would be true in the comprehensive sense, e.g., when considering all possible trajectories and model configurations, however, the utility of this information becomes more limited the farther out the prediction goes, as seen in Figure 5.

The model reseedings in Figure 6 indicate to use that the model is in a very deterministic configuration. Due to the spatial distribution of the injury, there is essentially no chance that it will heal the *in silico* patient back to full health, while also being in no danger of an immediate/near-term death. Essentially, the simulations are entirely under the influence of a single Probabilistic Basin of Attraction; this is discussed in detail in Cockrell and An (2017). Therefore, while the fully spatially realized simulation does not have a memory (and can safely be treated as a Markov process), the historical paths of the aggregate cytokine/health trajectories do provide some predictive ability; while information regarding the spatial distribution of tissue damage and systemic response is washed away when considering the trajectory of the system as a whole, features that describe the time evolution of the trajectory (i.e., temporal derivatives) play a role in the future predictions.

The failure to identify effective drugs to treat sepsis is due in significant part to a failure to account for the heterogeneity of the state space for sepsis and the non-uniqueness of mapping from state space to trajectory space: without understanding the potential future histories of an individual patient from any point in time there can be no rationally justified attempt at controlling or steering that patient’s eventual outcome. We have proposed that mechanism-based multi-scale computational models (as defined by the National Institutes of Health Interagency Modeling and Analysis Group^1^) can serve as proxy systems that can address the “Denominator Problem” that arises out of the non-uniqueness of the mapping between system state and behavior and the inevitable sparsity of biological data (An, 2018); we pose that the IIRABM represents one example of a proxy model for sepsis. However, for multiscale modeling and simulation to be deployed in clinical practice, it must be practical to utilize the models in a clinical setting. As we have shown in previous work (Cockrell and An, 2017), this requires an immense amount of computational power as the simulation must be repeated for many stochastic replicates. Compressing/approximating the information and dynamics contained within the computational model using an ANN allows for a computationally cheap and tractable method of rapidly updating predictions about patient disease trajectory as new information becomes available. Therefore, ability to predict requires an additional layer of surrogate models to render such prediction clinically tractable, and the complexity of the dynamic structure of inflammation/sepsis calls for the use of ANNs for this purpose. While there have been some attempts to use ANNs to serve as surrogates for multiscale models (Lagaris et al., 1998; Wang et al., 2019), these approaches involve the approximation of models that are based on known and explicitly described functions. Given that the Universal Approximation Theorem states that an ANN can be trained to reproduce any function, knowing the target function beforehand provides a greater likelihood of success. This is in direct contrast to ABM, which are generally explicitly used because they have no equivalent equation-based formulation. In particular, it is the ability to generate more biologically realistic probability distributions of behavior, as seen in Figure 1A and Ref (Cockrell and An, 2017). We posit that this is due to the nature of the noise in ABM’s compared to stochastic differential equation methods. In contrast to a differential equation, an ABM does not typically have closed form expression describing the randomness in the simulation; rather, randomness in the execution of the ABM is biologically motivated and incorporates aspects of observed biological heterogeneity (i.e., the spatial distribution of tissue resident macrophages in our prior sepsis simulations). Therefore, ANNs trained on ABMs inherently have a forecast horizon and prediction/forecasting applications of such ANNs need to account for updates of system state in order to provide a “rolling” forecasting cone. The concept is similar to that as seen in weather prediction, with the notable difference that in weather models the future uncertainty is due to deterministic chaos whereas in the ABM/biological system it is due to intrinsic aleatory stochasticity.

This current work the first step of the development of a workflow that integrates mechanism-based ABMs with ML in order to train predictive ANNs that can inform what sort of sensing technology and capabilities need to be developed in the real world. The demonstration of the non-unique mapping between system-microstate (in terms of cytokine profiles) and an overall metric of system macrostate (e.g., system health) suggests that data-centric attempts to develop predictive models, which at their root involve reverse engineering causal relationships between microstate and macrostate, are futile. We assert that it is only through mechanism-based, generative simulations that the sufficient density of time series data can be made available to parse the multiple trajectories that can arise from a particular system state. The basis for this assertion lies in the fact that the computational mechanistic model (as opposed to a data-driven statistical model) limits the future possibility space to one that can evolve from experimentally validated biological (microstate) mechanisms, whereas a statistical model with a sufficient number of terms can fit any data set arbitrarily well. While this space or the number of potential configurations a biological system can exist in is combinatorially/astronomically large, it is not infinite.

Our simulations also demonstrate the crucial role that aleatory stochasticity plays in the time evolution of these multi-agent/cellular systems, thereby necessitating a “rolling forecast” approach in which the update interval is informed by the simulations. Integrating mechanism-based, multi-scale simulations with ML provides a means of training predictive ANNs “off-line,” circumventing the need to run high-fidelity simulations in real time. Freed from the constraint of execution time, this in turn allows for more detailed mechanistic simulation models able to generate synthetic data that more closely matches that produced by the real world system, and, crucially, performed in a fashion that more comprehensively captures the range of biological heterogeneity seen in the clinical setting and has the ability to potentially falsify the model’s underlying structure (for a full description of this process see Ref (Cockrell and An, 2021)). We hope that this work can provide a starting point for additional investigations into the integration of ML and agent-based models.

## Data Availability Statement

The original contributions presented in the study are included in the article/supplementary material, further inquiries can be directed to the corresponding author.

## Author Contributions

RC and GA conceived the model and the experiments. DL performed the machine-learning work and analysis. All authors contributed to the manuscript.

## Author Disclaimer

The content of the information does not necessarily reflect the position or the policy of the Government, and no official endorsement should be inferred.

## Conflict of Interest

The authors declare that the research was conducted in the absence of any commercial or financial relationships that could be construed as a potential conflict of interest.

## Publisher’s Note

All claims expressed in this article are solely those of the authors and do not necessarily represent those of their affiliated organizations, or those of the publisher, the editors and the reviewers. Any product that may be evaluated in this article, or claim that may be made by its manufacturer, is not guaranteed or endorsed by the publisher.

## Footnotes

1
https://www.imagwiki.nibib.nih.gov/content/multiscale-modeling-msm-consortium
